# Supersalinity of Blood, Etc.

**Published:** 1882-07

**Authors:** 


### BOOK NOTICES, REVIEWS, ETC.

SUPERSALINITY OF THE BLOOD: AN ACCELERATOR OF SENILITY, AND A CAUSE OF CATARACT. By J. Compton Burnett, M. D. London: The Homœopathic Publishing Co., 2 Finsbury Circus, E. C. New York and Philadelphia: Bœricke & Tafel. 1882.

The accomplished editor of the *Homœopathic World* has given the profession several very interesting and practical monographs. Among these we may especially note those relating to the *Medicinal Treatment of Diseases of the Veins, the Prevention of Defect, Deformity and Disease,* and the *Curability of Cataract with Medicines.*

These monographs should be read and well pondered over by all. They furnish more food for thought than a dozen trashy allopathic works. And better yet, two of them prove to us that homœopathic medication *can* do much better for our patient than the knife.

This new work is perhaps more original in its scope than the other books of Dr. Burnett; and rather militates against the accepted physiological doctrine of the day, which teaches that salt is a necessary article of our diet. Nevertheless it is entirely reasonable in its tone and not at all carried away by too great enthusiasm for a pet notion, as one is apt to be. We would advise our readers to purchase all of Dr. B.'s monographs, for they will make a useful addition to one's library.

